# Analogs of the novel phytohormone, strigolactone, trigger apoptosis and synergize with PARP inhibitors by inducing DNA damage and inhibiting DNA repair

**DOI:** 10.18632/oncotarget.7414

**Published:** 2016-02-16

**Authors:** Michael P. Croglio, Jefferson M. Haake, Colin P. Ryan, Victor S. Wang, Jennifer Lapier, Jamie P. Schlarbaum, Yaron Dayani, Emma Artuso, Cristina Prandi, Hinanit Koltai, Keli Agama, Yves Pommier, Yu Chen, Lucas Tricoli, Jeannine R. LaRocque, Christopher Albanese, Ronit I. Yarden

**Affiliations:** ^1^ Department of Human Science, NHS, Georgetown University Medical Center, NW, Washington DC, USA; ^2^ The Lombardi Comprehensive Cancer Center, Georgetown University Medical Center, NW, Washington DC, USA; ^3^ Department of Chemistry, University of Turin, Turin, Italy; ^4^ Institute of Plant Sciences, ARO, Volcani Center, Bet Dagan, Israel; ^5^ Developmental Therapeutics Branch and Laboratory of Molecular Pharmacology, Center for Cancer Research, National Cancer Institute, NIH, Bethesda MD, USA; ^6^ Memorial Sloan Kettering Cancer Center, NY, NY, USA; ^7^ Department of Pathology, Georgetown University Medical Center, Washington DC, USA

**Keywords:** strigolactone, small molecule, homology-directed repair, RAD51, PARP inhibitors

## Abstract

Strigolactones are a novel class of plant hormones produced in roots that regulate shoot and root development. We previously reported that strigolactone analogs (SLs) induce G2/M cell cycle arrest and apoptosis in a variety of human cancer cells and inhibit tumor growth of human breast cancer xenografts in mice. SLs had no significant influences on non-transformed cells. Here we report for the first time that SLs induce DNA damage in the form of DNA double-strand breaks (DSBs) and activate the DNA damage response signaling by inducing phosphorylation of ATM, ATR and DNA-PKcs and co-localization of the DNA damage signaling protein, 53BP1, with γH2AX nuclear foci. We further report that in addition to DSBs induction, SLs simultaneously impair DSBs repair, mostly homology-directed repair (HDR) and to a lesser extent non-homologous end joining (NHEJ). In response to SLs, RAD51, the homologous DSB repair protein, is ubiquitinated and targeted for proteasomal degradation and it fails to co-localize with γH2AX foci. Interestingly, SLs synergize with DNA damaging agents-based therapeutics. The combination of PARP inhibitors and SLs showed an especially potent synergy, but only in BRCA1-proficient cells. No synergy was observed between SLs and PARP inhibitors in BRCA1-deficient cells, supporting a role for SLs in HDR impairment. Together, our data suggest that SLs increase genome instability and cell death by a unique mechanism of inducing DNA damage and inhibiting DNA repair.

## INTRODUCTION

Cancer remains the second-leading cause of death in the United States. Treatment of advanced disease with classical chemotherapeutic drugs is completely successful in only several types of cancer such as testicular carcinoma and childhood leukemia [1]. In most other types of cancer, despite an initial favorable response, local and distant relapses associated with resistance to chemotherapy invariably occur and thus result in limited survival benefits [1]. Therefore, the development of safe and effective drugs along with novel therapeutic strategies that successfully eradicate tumors and metastatic growth are highly desirable. A challenge arises in identifying therapeutic combinations that will target both the hyperproliferative cells as well as the slow-growing cancer stem-like cells that are capable of self-renewal and survival after therapy. Moreover, productive combinations may create synergistic responses permitting the use of the lowest possible drug dosages to effectively target all cancer cells and reduce toxic side effects [1-3].

Strigolactones are a novel class of phytohormones produced by a wide variety of plant species [4]. In plants, Strigolactones are involved in pleiotropic effects including endogenous control of above ground plant development *via* bud outgrowth repression and inhibition of shoot meristem [5]. Previously, we reported that small molecules, synthetic analogs of SLs, induce G2/M arrest and apoptosis in a variety of human cancer cells, but have minimal influence on growth and viability of non-transformed human fibroblasts, mammary epithelial cells, as well as normal primary prostate cells [6, 7]. *In vivo*, SL analogs inhibit the growth of human MDA-MB-231 xenografts [8]. Interestingly, cancer cells with stem-like properties are more sensitive to the inhibitory effects of SLs analogs than the heterogeneous population of cancer cells [6].

We showed that SLs' inhibitory effects are associated with activation of the stress-activated MAPKs, P38 and JNK1/2, interference with the tubulin network, and inhibition of the survival factors, ERK1/2 and AKT [6-8]. Our current knowledge as to the exact mechanism of action, however, is limited. The activation of the stress response led us to hypothesize that SLs induce DNA damage and genomic instability. Double-strand breaks (DSBs) are considered the most deleterious form of DNA damage and DSBs repair is essential for tumor cell survival [9, 10]. There are two major pathways of DSBs repair: an error-prone non-homologous end joining (NHEJ) that often includes processing of the DNA broken ends before ligation, and the error-free homology-directed repair (HDR) that uses a homologous sequence (most often a sister chromatid) as a template for repair [11-13]. While cells employ NHEJ throughout the cell cycle, HDR is utilized only during S and G2 phases [12]. Cells with compromised HDR are prone to synthetic lethality when triggered by DNA-damaging agents. This has been exemplifed in BRCA1- and BRCA2-mutated tumors treated with PARP inhibitors [14-18] or platinum-based chemotherapy [14, 19, 20]. RAD51 is another critical component of the HDR machinery. RAD51 assembly onto single-stranded DNA ends catalyzes the exchange of homologous DNA sequences at the break sites [10, 21]. Suppression of RAD51 sensitizes cancer cells to DNA-damaging drugs [10, 22-25], and RAD51 overexpression contributes to chemotherapy resistance in several types of cancer including breast, prostate and human soft tissue sarcoma cells [10, 26-31].

In the present study, which confirms the selective anti-proliferative effect of SLs in cancer cells, we establish that SLs induce DSBs and inhibit their repair by HDR and NHEJ. We further show that SLs down regulate the expression of RAD51 in a proteasome-dependent manner and inhibit RAD51 re-localization to DSB sites. Together, our studies suggest that by inducing a “BRCAness” phenotype, SLs can sensitize cells to chemotherapeutic drugs such as PARP inhibitors to enhance their efficacy.

## RESULTS

### Strigolactones induce apoptosis in U2OS osteosarcoma cells

**Figure 1:**
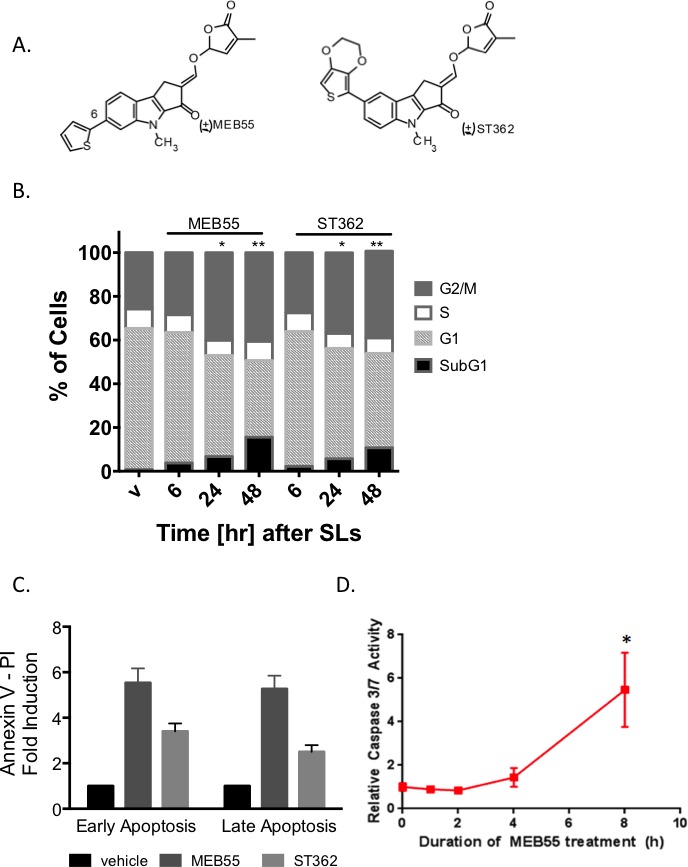
Strigolactone analogs induce G2/M arrest and apoptotic cell death **A.** The chemical structure of synthetic analogs of strigolactone MEB55 (1 ppm = 3.1 μM) and ST362 (1 ppm = 2.1 μM) **B.** U2OS cells were treated with MEB55 or ST362 at 10 ppm for 6, 24, and 48 hr, or with DMSO vehicle (V). Graph is representative of mean of three independent experiments. Cell cycle analysis was performed by flow cytometry (* *p* < 0.05; ** *p* ≤ 0.001). **C.** U2OS cells treated with MEB55 or ST362 at 10 ppm for 24 hr were stained with Annexin V/PI and analyzed relative to vehicle (DMSO)-treated cells by flow cytometry. Graph is representative of mean ± SD of at least three independent experiments. **D.** Caspase activation, as measured by Caspase-3/7 Glo luciferase assay of U2OS cells treated with MEB55 at 10 ppm for the indicated durations, relative to 24 hr vehicle-treated (DMSO) control cells. Graph is representative of mean ± SD of at least three independent experiments. (* *p* < 0.05; ** *p* ≤ 0.001).

We previously reported that synthetic analogues of strigolactones (SLs) (Figure 1A) reduce the viability of a variety of cancer cells [6, 7]. To assess whether SLs induce cell cycle arrest or cell death in the U2OS osteosarcoma cell line, we stained the cells with propidium iodide (PI) and examined cell cycle progression by FACS. A significant increase in the percentage of U2OS cells in G2/M and sub-G1 phases was detected at 24 and 48 hr after treatment with the strigolactone analogs MEB55 or ST362 (Figure 1B). To further assess the impact of SLs on cell death, dual staining with Annexin V and PI was employed. Annexin V is a marker of early apoptosis, while PI stains cells in late apoptosis with a compromised plasma membrane. A significant increase in early and late apoptosis was detected in U2OS cells after 24 hr treatment with either MEB55 or ST362 (Figure 1C and Supplementary Figure S1). An increase in caspase 3/7 activity was detected as early as 8 hours after treatment with MEB55 and further corroborated the induction of MEB55-mediated apoptotic cell death (Figure 1D).

### Strigolactones induce genomic instability and DNA double-strand breaks

**Figure 2:**
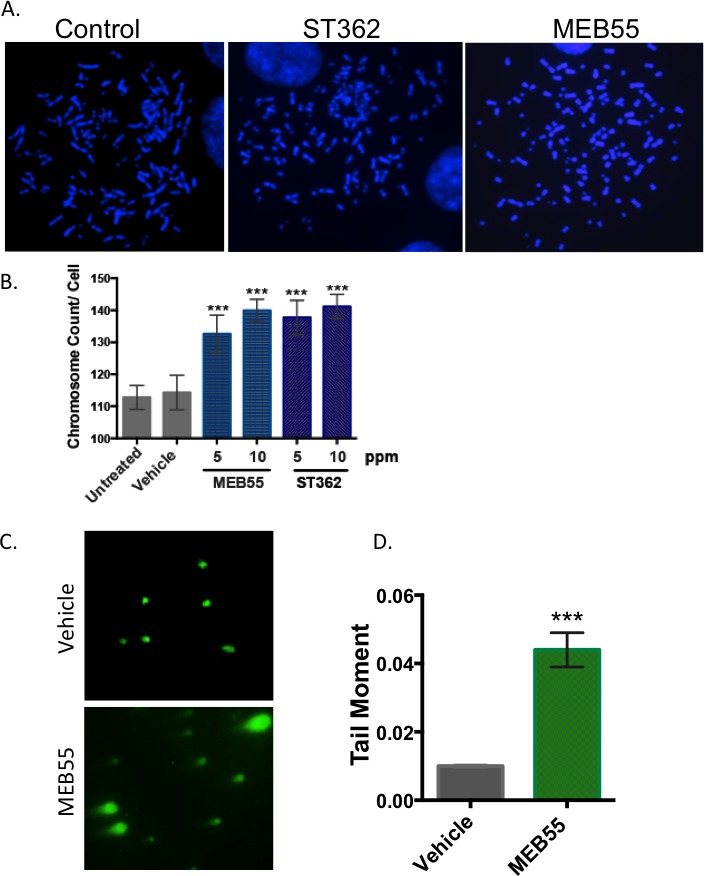

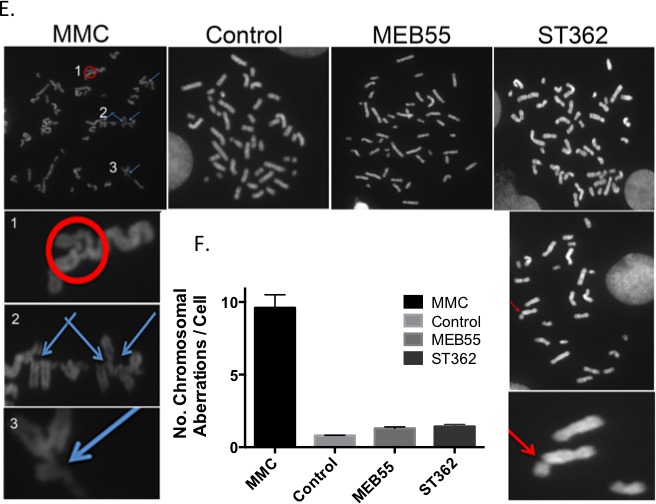
SLs induce DSBs and increase genomic instability **A.** U2OS cells were treated with 5 or 10 ppm of MEB55 or ST362 for 5 hr and subjected to metaphase spread assays to examine chromosome integrity. Representative images from at least 50 images are shown. **B.** Quantitative analysis of chromosomal breaks. The number of chromosomes per spread was counted from at least 50 spreads for each sample. The experiment was repeated at least three times and the data represent mean ± SD. (* *p* < 0.04; ** *p* ≤ 0.0004). **C.** U2OS cells were treated with vehicle (top panel) or MEB55 at 10 ppm (bottom panel) for 5 hr. The presence of DSBs is indicated by the Neutral Comet assay. **D.** Quantitative analysis of at least two independent experiments. At least 30 cells were scored. Values represent mean ±SD. (*** *p* < 0.0001). **E**. FANCC cells were treated with either vehicle control, 300 nM MMC or SLs at 10 ppm for 72 hrs and subjected to metaphase spreads. Representative images from at least 50 spreads are shown. **F**. The number of chromosomal aberrations in response to the different treatments (crosslinking and breakage) were quantified. Values represent mean +/− SD. (** *P*<0.001)­­­­.

Next, we examined the possibility that cellular responses to SLs are associated with DNA damage and loss of genomic stability. Metaphase spreads of U2OS cells were prepared and stained with DAPI after treatment with 5 or 10 ppm of MEB55 or ST362 for 24 hr. U2OS osteosarcoma cells contain chromosome counts in the hypertriploid range; the average chromosome count in untreated cells displaying long intact chromosomes (Figure 2A) is 113±7.3 chromosomes per metaphase spread. Treatments with MEB55 or ST362 led to a significant increase in chromosome count (Figure 2A) with an average of 140±3.4 and 141±3.8 chromosomes per metaphase spread, respectively (***p < 0.0001) (Figure 2B).

The induction of DNA DSBs in SLs-treated cells was verified using the neutral Comet Assay, an electrophoretic method to measure DNA damage and the presence of DNA DSBs. Vehicle and MEB-treated cells at 10 ppm were harvested after 4 hr and visualized on Comet slides using SYBR green. The presence of “tails” projecting from the embedded cells indicates damaged DNA fragments that have migrated farther during electrophoresis. Images taken of the vehicle and treated cells indicate an increased presence of tails in the MEB55-treated cells (Figure 2C). Quantification of this observation using the CometScore program indicated a 4-fold increase in tail moment in strigolactone-treated cell samples (***p < 0.0001) validating that SLs induce DSBs (Figure 2D).

To determine whether SLs induce chromosome breakage *via* DNA crosslinking, we analyzed the chromosomes of lymphoblast cells derived from a Fanconi Anemia complementation group C (FANCC) patient. Metaphase analysis demonstrates that the FANCC cells are highly sensitive to mitomycin C (MMC), a DNA crosslinking agent, that induces both chromosome crosslinking and DNA breaks. SLs caused a higher rate of chromosome breaks relative to control (1.4 breaks per cell in ST362-treated cells *vs*. 0.8 breaks /cell in control cells) (Figure 2E and 2F). However, no chromosomal crosslinking was observed in SL-treated cells (Figure 2E).

### Strigolactone treatment leads to activation of the DNA damage response in U2OS cells

**Figure 3:**
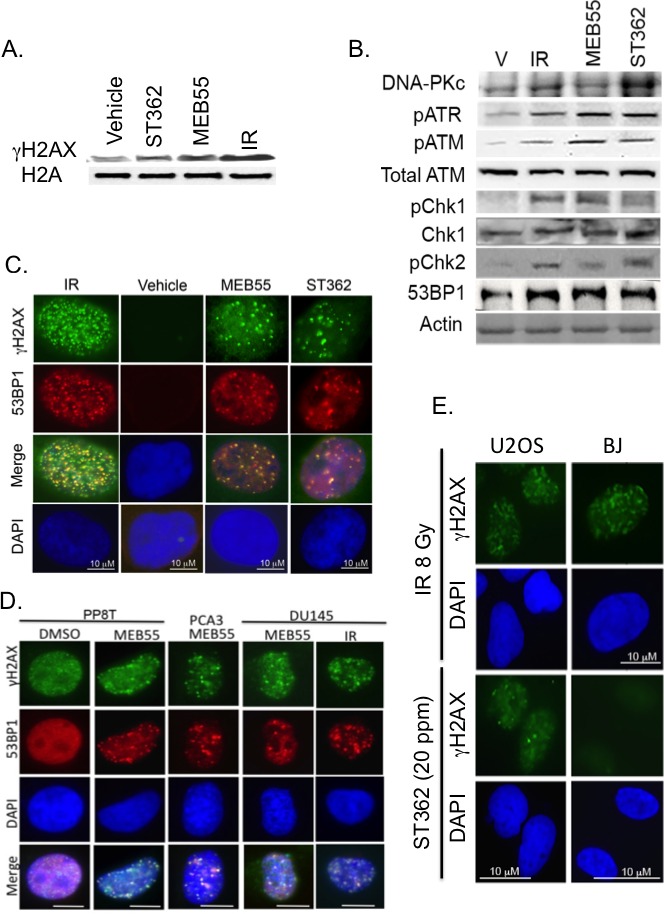
SLs activate the DNA damage response-signaling cascade **A.** U2OS cells were treated with vehicle or 10 ppm of SLs (MEB55 or ST362) for 4 hr. Following histone extraction, expression levels of histone H2A and γH2AX were analyzed by immunoblot. **B.** Following treatment with 10 ppm of MEB55 or ST362, U2OS cells were lysed and expression levels of ATM, phosphorylated ATM, ATR and DNA-PKcs as well as phosphorylated Chk1 and Chk2 were analyzed by immunoblot. β-Actin was employed as a loading control. **C.** U2OS cells were treated with 10 ppm of ST362 or MEB55 for 3 hr or with 8 Gy ionizing radiation (IR) as a positive control. Immunofluorescent analysis indicates that 53BP1 co-localizes with γH2AX foci. **D.** Co-localization of 53BP1 and γH2AX in nuclear foci in DU145 prostate cancer cell line, patient-derived conditionally-reprogrammed prostate cancer cells (PP8T), or patient-derived prostate cancer organoid conditionally-reprogrammed PCA3 cells treated with 10 ppm of MEB55 or IR as positive control. **E.** γH2AX foci in U2OS and BJ fibroblasts in response to 8 Gy IR or 20 ppm ST362.

To determine whether SLs treatment elicits activation of the DNA damage response (DDR), we analyzed the phosphorylation of the DNA DSBs marker, histone H2AX [32], in U2OS cells treated with 10 ppm of MEB55 or ST362 for 4 hr. Figure 3A shows that expression of γH2AX is induced in SLs-treated cells similar to cells exposed to 8 Gy of ionizing radiation (Figure 3B). We further analyzed the phosphorylation/activation of the DNA damage sensors ATM, ATR, DNA-PKcs and their downstream checkpoint kinases, Chk1 and Chk2 [33-35]. U2OS cells were treated with either MEB55 or ST362 at 10 ppm for 4 hours or with 8 Gy of ionizing radiation (IR) as a positive control. Immunoblot analysis revealed that ATM, ATR and DNA-PKcs phosphorylation is induced in response to SLs, in addition to the phosphorylation of Chk1 and Chk2 kinases (Figure 3B and Supplementary Figure S2). Moreover, we show that the well-established DSB marker, 53BP1, is recruited to DSB sites and co-localizes with γH2AX foci [36, 37] upon treatment with 10 ppm of ST362 (Figure 3C and supplementary Figure S3) or 10 ppm of MEB55 (Figure 3D).

53BP1 is also recruited to sites of DSBs together with γH2AX in DU145 prostate cancer cells, in a castration-resistant patient-derived organoid line termed CRPC-PCA3 [38] [7, 39, 40, 41], and in a patient-derived CRC line PP8T, from a patient diagnosed with Gleason's score 8 prostate cancer (Figure 3E). Conversely, SLs failed to elicit γH2AX foci and DSBs in non-transformed BJ fibroblast cells (Figure 3E and Supplementary Figure S4).

### Strigolactone analogs interfere with RAD51 expression and co-localization to DSBs

**Figure 4:**
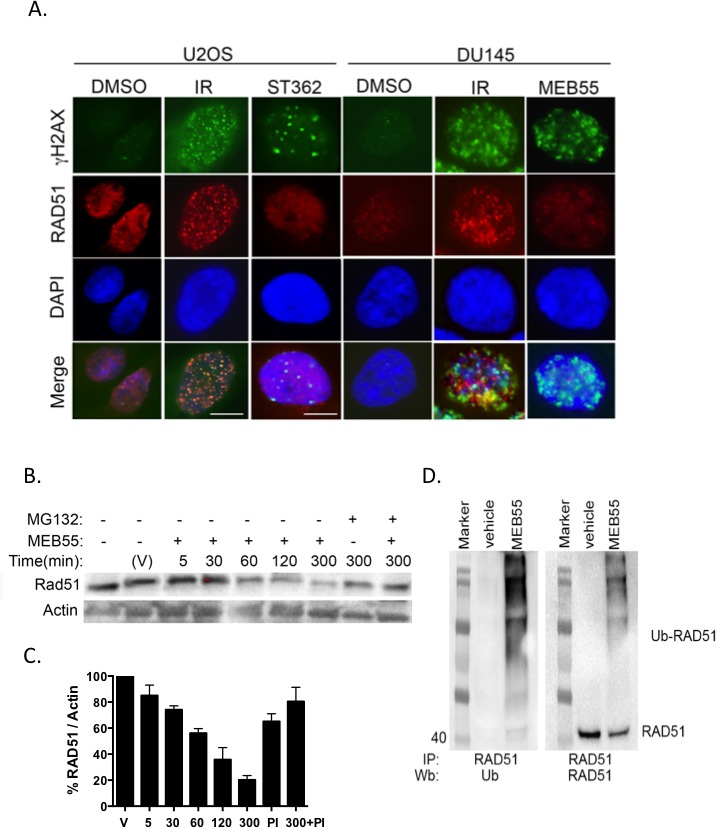
SLs affect RAD51 expression and function **A.** RAD51 fails to co-localize with γH2AX foci. U2OS and DU145 cells were treated with ST362 or MEB55 at 10 ppm for 4 hr or with 8 Gy IR as a positive control and then stained for immunofluorescent analysis of RAD51 and γH2AX. **B.** SLs down regulate RAD51. U2OS cells were treated with 10 ppm of MEB55 for the indicated times or pre-treated with MG132 at 10 μM 1 hr prior to MEB55 treatment. RAD51 expression was analyzed by immunoblot. β-Actin was employed as a loading control. The experiment was repeated at least three times. **C.** Quantification of normalized RAD51 relative to vehicle-treated cells. **D.** RAD51 is poly-ubiquitinated upon SLs-treatment. U2OS cells were pre-treated with 10 μM MG132 1 hr prior to treatment with 10 ppm of MEB55 or vehicle. Three mg of whole cell lysates were incubated overnight at 4°C with a polyclonal antibody against RAD51 and protein A/G. Ubiquitination was resolved by immunoblot with an antibody against poly-ubiquitined proteins (left panel) or RAD51 (right panel).

We next analyzed whether the homologous repair protein, RAD51, co-localizes to the DSBs sites formed in response to SLs treatment [21, 34]. RAD51 recruitment to γH2AX sites indicates that cells have initiated DNA repair by HDR at the damaged sites [34]. Because SLs cause cell cycle arrest at the G2/M phase [6, 7], we hypothesized that HDR would be the main mechanism of DSBs repair. However, unlike 53BP1, RAD51 did not form distinct foci that co-localize with γH2AX foci in U2OS cells or in DU145 cells treated with 10 ppm MEB55 or ST362 (Figure 4A). Moreover, immunoblot analysis revealed that RAD51 expression is down-regulated beginning 1 hr after treatment with 10 ppm of MEB55 and continues to decline by 5 hr. Pre-treatment of cells with 10 μM of the proteasome inhibitor, MG132, alleviated RAD51 down-regulation by SLs (Figure 4B and 4C) and demonstrated that RAD51 becomes poly-ubiquitinated following SLs treatment (Figure 4D). Together, our data indicate that SLs target RAD51 for proteasomal degradation, thereby disrupting its redistribution onto sites of DSBs.

### Strigolactones inhibit DSB repair

The failure of RAD51 to co-localize with DSB sites after SLs treatment led us to hypothesize that DSBs repair is impaired in SLs-treated cells, which could be the mechanism leading to SLs-mediated cell death. DSBs can be repaired by homology-directed repair (HDR) or by non-homologous end joining (NHEJ) [11, 13]. We utilized two well-characterized GFP-based reporter systems to gain insight into the mechanisms by which SLs inhibit DSBs repair. The HDR assay was based on U2OS cells that stably carry the direct repeat (DR)-GFP construct [42].

**Figure 5:**
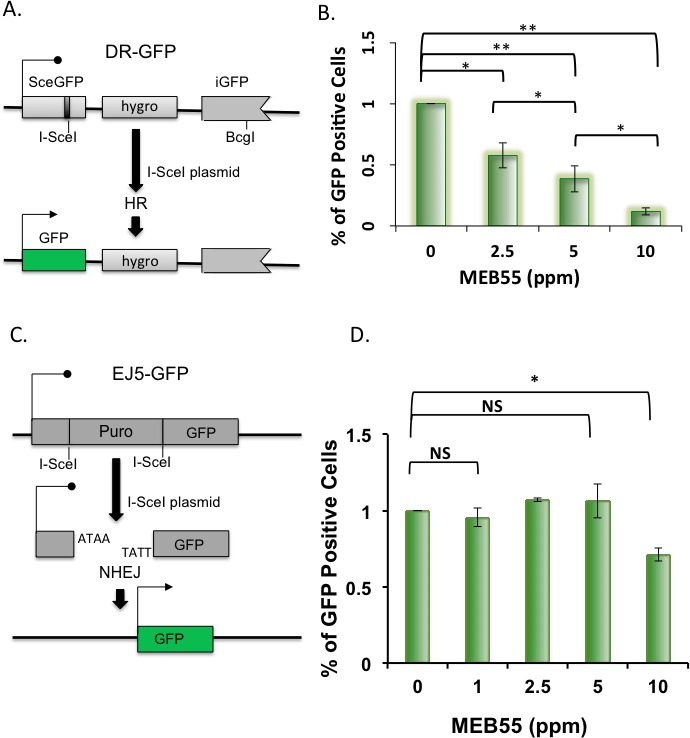
SLs impair Homology-Directed Repair of DNA DSBs **A.** A schematic representation of the DR-GFP reporter assay. **B.** U2OS cells stably carrying the DR-GFP reporter were transfected with I-*Sce*I expression plasmid and on the following day were treated with the indicated concentrations of MEB55 for 24 hr. GFP positive cells were quantified 24 hr later by FACS. Results represent mean ± SD from at least three independent experiments. **C.** A schematic representation of the EJ5-GFP reporter assay. **D**. U2OS cells stably carrying the EJ5-GFP reporter were transfected with I-*Sce*I expression plasmid and on the following day were treated with the indicated concentrations of MEB55 for 24 hr. GFP positive cells were quantified 24 hr later by FACS. Results represent mean ± SD from at least three independent experiments.

Following transfection of the I-SceI restriction enzyme expression plasmid (pCBAS), which cleaves the DR-GFP reporter construct, cells were treated with different concentrations of MEB55 for 24 hr. GFP fluorescence occurs only if the cell regains a complete GFP sequence by HDR as determined by FACS analysis (Figure 5A). A significant dose-dependent decrease in GFP fluorescence was observed following MEB55 treatment as compared to control, vehicle-treated, cells (Figure 5B). Each concentration of MEB55 (2.5, 5, and 10 ppm) showed a statistically significant decrease of about 42%, 60% and 90 % in GFP+ cells as compared with the control cells (**p ≤ 0.01, and ***p ≤ 0.0004, respectively). Given these results, we conclude that MEB55 inhibits the frequency of HDR in cancer cells, the preferable DSB repair pathway when cells are arrested in G2 phase. Interestingly, a previous report by Goldstein and Kastan also showed a 90% decrease in HDR upon RAD51 depletion [43].

Next, we utilized a parallel reporter system to analyze the effect of MEB55 on NHEJ. The EJ5-GFP reporter construct is stably integrated into U2OS cells [44]. The reporter consists of two I-SceI sites flanking a puromycin resistance gene. Following transfection of the I-SceI expressing plasmid, pCBAS, the *puro* gene is excised upon the repair by NHEJ, allowing for recovery of the GFP gene and GFP expression (Figure 5C). EJ5-GFP cells transfected with pCBAS were treated with increasing concentrations of MEB55. At low concentrations (1-5 ppm), MEB55 had no effect on NHEJ activity relative to vehicle-treated cells; MEB55 at 10 ppm, however, caused a 30% decrease in NHEJ activity (*p ≤ 0.025), suggesting that the NHEJ repair pathway is also compromised (Figure 5D). Overall, these results suggest that SLs impair HDR and NHEJ repair mechanisms.

### Strigolactones do not intercalate into DNA, but sensitize cells to PARP inhibitors

**Figure 6:**
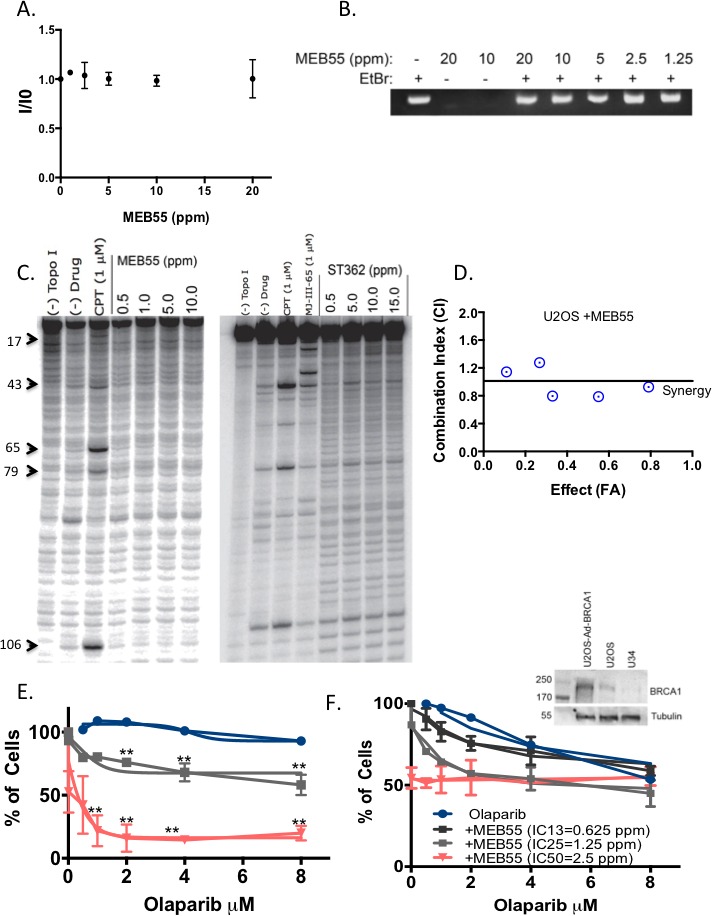
SLs synergize with PARP inhibitors to reduce U2OS cancer cell viability **A.** SLs do not intercalate into DNA and do not displace EtBr. Circular DNA was incubated with EtBr at 0.8 ug/mL for 1 hr before the indicated concentrations of MEB55 were added. The fluorescence intensity (I/I0) of samples was excited at 525 nm, and measured at 590 nm. **B.** Similar to (A) except DNA was resolved on a 0.8% agarose gel. **C.** Topoisomerase-I inhibition assays. The purified, end-labeled 117 bp nucleotide encompassing Top1 cleavage sites was incubated with recombinant Top1 enzyme in the presence of various concentrations of MEB55 (left) or ST362 (right). **D.** U2OS cells were seeded in 6 replicates into 96-well plates (2500/well) and their growth in response to 96 hr treatments with different concentrations of MEB55 and doxorubicin relative to vehicle-treated (DMSO) cells was analyzed by XTT assay. C-Index curve analysis (Chou and Talalay plot [46]) for DOX/SLs in U2OS indicates synergy [46] when C-Index values are less than 1 (the horizontal line that indicates the additive effect). Values greater than 1 indicate antagonism. To calculate the FA point DOX/SLs were mixed using constant ratios corresponding to 0.25, 0.5, 1, 2, and 4 times the IC_50_ for each drug. Graph is representative of at least three independent experiments. **E.** U2OS cells were seeded into 96-well plates (2500/well) in 6 replicates and their growth in response to 96 hr treatments with different concentrations of MEB55 and olaparib relative to vehicle-treated (DMSO) cells was analyzed by crystal violet assay. Graph is representative of mean ± SD from at least three independent experiments. Data points are connected by non-linear regression of the sigmoidal dose-response. The IC_50_ value of olaparib in the presence of MEB55 is 0.329 μM with 95% CI (1.121-5.356). **F.** BRCA1-silenced U2OS, U34, [51] growth in response to MEB55 and olaparib was analyzed as in (E). An immunoblot is presented to confirm the loss of BRCA1 expression in U34, relative to parental U2OS and U2OS cells overexpressing BRCA1 as previously described [61].

To further elucidate the mechanisms by which SLs cause DSBs and interfere with their repair, we tested whether SLs intercalate into the DNA by an ethidium bromide (EtBr) displacement assay [45]. The fluorescence of EtBr is weak in aqueous solution, but its strong intercalation into the DNA greatly enhances its fluorescence intensity. Using cell-free circulating DNA (CT-DNA), we tested whether SLs can displace pre-bound EtBr. Figure 6A shows that despite increasing concentrations (1.25 ppm-20 ppm) of MEB55 or ST362, there was no decrease in the emission intensity of EtBr, indicating that SLs do not displace or compete with EtBr (Figure 6A, 6B and Supplementary Figure S5).

We next tested for additive or synergistic effects on growth inhibition when SLs were combined with doxorubicin (DOX), which intercalates into the DNA and causes DNA damage by inhibiting DNA topoisomerase II. The IC_50_ of DOX in U2OS cells (1.72 μM) was determined by XTT assay after 72 hr treatment (Supplementary Figure S6). Based on the method of non-constant ratio drug combination proposed by Chou and Talalay [46], XTT viability assays were carried out in non-constant ratios corresponding to 2 concentrations below and above the IC_50_ for each compound (Supplementary Figure S7). Combination index (C-Index) analyses of DOX and SLs were performed using the CompuSyn software and were based on the C-Index equation [46]. The effect levels (Supplementary Table 2) and data in Figure 6D illustrate C-Index values below (synergy), and above (antagonism) the 1 additivity line. Based on the C-Index values less than 1, partial synergy can be seen in three different combinations between DOX and MEB55 when concentrations of both compounds are below their IC_50_. In addition, SLs did not induce the typical DNA cleavage pattern produced by camptothecin or MJ-III-65, both of which are DNA topoisomerase I inhibitors (Figure 6C) [47, 48]. Taken together, these results indicate it is unlikely that SLs cause DSBs through DNA intercalation and inhibition of topoisomerase I or II enzymes.

Because cells deficient in HDR are sensitive to PARP inhibitors [9, 14], we tested whether SLs-treated cells with reduced RAD51 and HDR efficiency exhibit enhanced sensitivity to the commercially available PARP inhibitors (olaparib and veliparib). As expected, olaparib itself (with increasing concentrations up to 8 μM) did not affect U2OS cell viability, and veliparib alone affected cell viability only slightly at the highest concentrations of 40 μM. In contrast, the survival fraction of cells treated with 2.5 ppm of MEB55 (just below the IC_50_ concentration: of 2.7 ppm) alone was 60% ± 0.6 and together with 8 μM olaparib was reduced to 38% ± 1.0 (*p* ≤ 0.007). Similar results were obtained even in the presence of lower MEB55 concentrations (1.25 ppm, equivalent to IC_23_), at which the survival fraction of SLs-treated cells was 76% ± 6.6 and in combination with 8 μM olaparib was further reduced to 53% ± 1.7 (*p* ≤ 0.005) (Figure 6E). Non-linear regression analysis of the sigmoidal dose response revealed that IC_50_ values of olaparib were significantly reduced to 0.329 μM with 95% confidence interval (CI) (1.121-5.356 *p* ≤ 0.001, in the presence of MEB55 at 2.5 ppm.

The synergy between SLs and PARP inhibitors was calculated according to Kern, *et al*. [49] and Romaelli, *et al*. [50]. This method is suitable when one compound has no cytotoxic effect of its own. According to this method, the R index above 1 (1 = additive effect) indicates synergy. An R index of 2.7 was observed when cells were treated with the combination of olaparib and MEB55 (2.5 ppm). An R index of 2.2 was observed when cells were treated with veliparib and MEB55 (Supplementary Figure S8). At 20 μM veliparib, the survival fraction of cells was reduced to 28.5% ± 9.3 (*p* ≤0.01) and was further reduced to 18% ± 9.3 with 40 μM of veliparib (*p* ≤ 0.007). A similar synergy was also detected when the triple negative, breast cancer cells, MDA-MB-231, were treated with the combination of veliparib and MEB55 (Supplementary Figure S9). The combination of PARP inhibitors with SLs is highly synergistic with a remarkable loss of cell viability relative to each individual treatment. As expected, our previously published U34 cells (U2OS cells that are stably silenced for BRCA1) [51], are sensitive to olaparib. The R index for olaparib and MEB55 (IC_50_) is 0.56; for MEB55 (IC_25_) R = 0.72 and for MEB55 IC_13_ R = 1.0, indicating that MEB55 (IC_50_) combination with olaparib does not provide further sensitivity; an R index of 0.56 suggests that SLs (MEB55) target the HDR pathway and that BRCA1 is epistatic to SLs target.

## DISCUSSION

The results of this study show that the synthetic strigolactone analogs, MEB55 and ST362, induce G2/M cell cycle arrest and apoptosis of cancer cells [6, 7], which is accompanied by induction of DNA damage in the form of DSBs and inhibition of DSB repair. The increase in chromosome count following SLs treatment was visualized by metaphase spreads. The presence of DNA DSBs was visualized by the non-denaturing Comet assay and was further supported by the formation of nuclear γH2AX foci that mark DSBs. The phosphorylation of ATM and ATR kinases and their downstream effectors Chk1 and Chk2, as well as co-localization of 53BP1 with γH2AX foci indicate that cells sense SLs-mediated DNA damage and activate the DNA damage response signaling. The failure of the homologous recombination repair protein, RAD51, to localize to γH2AX-marked damage sites and activate HDR indicates that SLs induce uncoupling between DNA damage signaling and DNA repair that could trigger cell death. The selectivity of SLs toward cancer cells was demonstrated by the very few nuclear γH2AX foci indicative of DSBs that were detected in normal BJ fibroblasts with no significant differences between SLs-treated and non-treated cells.

DSBs are the most deleterious form of DNA damage. Once DSBs are formed, cells can utilize either one or both of the two main pathways for DSBs repair: an error prone non-homologous end joining (NHEJ) or an error-free homology-directed repair (HDR) [11, 52]. HDR is critical for cell survival but the activity is restricted to cells in late S or G2 phases of the cell cycle. On the other hand, NHEJ is utilized by cells throughout the cell cycle [13, 52]. Here we report that HDR is significantly inhibited in a dose-dependent manner by SLs in cancer cells. Interestingly, NHEJ repair initially failed to be activated relative to untreated cells despite an increase in DNA-PKcs phosphorylation and the presence and persistence of DSBs. As SLs induce the G2/M arrest [6, 7], we speculate that the substantial loss of HDR has greater impact than loss of NHEJ on cell fate and SL-induced cell death. We propose a mechanism for sensitivity of cells to SLs due to defects in both HDR and NHEJ repair.

Our current understanding of the molecular effects and targets of SLs is still limited. Our initial hypothesis that SLs could function as topoisomerase inhibitors based on their ability to induce DNA damage and inhibit DNA repair is not supported by our findings. DOX, one of the most widely used drugs in chemotherapy regimens, induces DNA DSBs by intercalating into the DNA and inhibiting DNA topoisomerase II. The interaction between SLs and DOX suggests that they do not act in a similar fashion. Furthermore, our direct biochemical assays clearly show that SLs do not intercalate into the DNA and do not inhibit topoisomerase activities.

Our metaphase analyses show elevated chromosome counts in cancer cells treated with SLs. Chromosome breakage was observed in the highly sensitive FANCC cells; however, there was no indication for induction of DNA crosslinking that was further corroborated by metaphase spread analyses of FANCC cells. Overall, our data suggest that SLs may cause DSBs, impair HDR, and induce cancer cell death through a unique mode of action.

Recent findings demonstrate that cancer cells and tumors deficient in HDR are highly sensitive to DNA damaging agents and PARP inhibitors by a mechanism of synthetic lethality [9]. This property has been successfully translated into the clinic for breast and ovarian cancer patients with mutations in the tumor suppressor genes, BRCA1 or BRCA2 [14, 53]. Due to HDR defects, these patients at least initially respond very well to PARP inhibitors [14].

RAD51 is an integral component of the cellular DNA damage response and a key factor for HDR [21, 34]. Its recruitment to DSB sites marked by γH2AX depends on BRCA2 and BRCA1 [54] and the quantification of RAD51 foci is well accepted as a marker of HDR activity [21, 34]. RAD51 expression is elevated in many human cancers including breast, prostate, lung and soft tissue sarcoma [25, 29, 30, 55]. RAD51 overexpression is associated with an increase in DNA repair activity that could account for tumor's resistance to radiotherapy and DNA damaging chemotherapy [10, 25, 26]. Thus, targeting RAD51 could increase the effectiveness of cancer treatments. While disease-associated mutations in RAD51 are extremely rare, depletion by siRNA or miRNA induces a “BRCAness” phenotype and sensitizes cancer cells to DNA damaging agents such as cisplatin and PARP1 inhibitors similarly, to BRCA1 and BRCA2 mutations [18, 23-25, 56].

Here we show a lack of distinct RAD51 nuclear foci in U2OS and DU145 cells despite the formation of γH2AX foci following SLs treatment. Furthermore, we demonstrate that RAD51 levels are down regulated by SLs *via* ubiquitination and proteasomal degradation, similar to the effect by another recently identified RAD51 inhibitor, IRB2 [22]. These findings imply that SLs target directly or indirectly RAD51 expression and function by interfering with RAD51 recruitment to DSB sites and hence with HDR. Given that BRCA1 is an E3 ubiquitin ligase activated by DNA damage [51], it would be interesting to identify the role SLs might play in modulating BRCA1 activity. Not surprisingly, our data show significant synergy between SLs and the PARP inhibitors, which inhibit base excision repair, in effectively killing U2OS cells. However, no synergy between SLs and PARP inhibitors was detected in the absence of BRCA1 expression, further supporting a role for SLs in HDR inhibition. Notably, SLs do not induce DNA damage nor cause cell death in normal, non-transformed human fibroblasts, suggesting that SLs may be of important clinical relevance. The selectivity of SLs to cancer cells may result from a differentially expressed, yet unknown receptor for SLs in cancer cells. Alternatively, the genomic instability already present in cancer cells may render them hypersensitive to the DNA damaging effect of SLs.

An important question that remains to be answered is whether SLs actions in mammalian cells resemble their mechanisms of action in plant cells. The receptor for SLs in plants was recently characterized as a heterodimer of an α/β-fold protein and an F-box protein (MAX2-More Axillary Growth 2). The F-box protein is a component of the SCF E3 ubiquitin ligase machinery and is responsible for recognizing and interacting with different substrates to result in their proteasomal degradation. Both proteins are present in the nucleus and the cytosol, and downstream events were recorded in both subcellular compartments [5, 57]. In mammalian cells, the cellular reception of SLs is still unknown. Our previous studies suggested that SLs-mediated signaling occurs at least partially in the nucleus as they are capable of altering cells' transcriptional program [7] and can influence the expression and activity of both nuclear and cytoplasmic proteins [6, 7, 58]. It is intriguing to speculate as to whether RAD51 is a ubiquitination target of the SLs receptor. We are currently using biotinylated fluorescent SLs to identify the mammalian SLs receptor and their cellular destination.

Finally, despite a large number of studies describing multiple signaling pathways that strigolactones utilize to regulate root and shoot development [59, 60], there are no reports on the role of strigolactones in maintenance of plant genome stability. It would be especially interesting to determine whether strigolactones suppress adventitious-root formation or the outgrowth of pre-formed axillary buds in shoots *via* induction of DNA damage and manipulation of DNA repair as in cancer cells.

## MATERIALS AND METHODS

### Cell culture, reagents and transfections

The human cell lines U2OS, DU145, MDA-MB-231 and BJ fibroblasts were obtained from ATCC and Georgetown University tissue culture core facility. U2OS-DR-GFP cells (a gift from M. Jasin, Memorial Sloan Kettering Cancer Center), were propagated in DMEM supplemented with 10% FBS. U2OS-EJ5-GFP cells (a gift from Dr. Jeremy Stark, City of Hope) were propagated as described [44]. All tissue culture media and serum were purchased from Gibco (Invitrogen), unless otherwise indicated. FANCC cells (GM13020) were purchased from Coriell Institute for Medical Research and maintained in suspension in RPMI-1640 supplemented with 15% FBS (HyClone Laboratories).

Human prostate samples were collected under the auspices and approval of the Georgetown University and Sloan Kettering Institutional Review Boards. For PP8T cells, pathological analysis confirmed that all tissue sections collected were nearly exclusively tumor cells. The specimens were processed *via* protease dissociation as previously described [7, 39]. Primary cultures were established at Georgetown University Medical Center using the CRC method as previously described [7, 38, 39]. For PCA3, cells from a prostate cancer organoid [38] were conditionally reprogrammed and co-cultured in F-media supplemented with Y27632 and with irradiated J2 fibroblasts. For SL studies, the cells were cultured in conditioned media as described [41]. Briefly, irradiated J2 feeder cells were plated in 175 cm^2^ tissue culture flasks (BD Biosciences, Franklin Lakes, NJ) in 30 ml of F media. The media was collected after three days in culture and centrifuged at 1000 *g* for 5 minutes at 4^°^C to remove cellular debris, followed by filtration using a 0.22 μm Millex-GP filter unit (Millipore, Billerica, MA). For cell culture, three volumes of conditioned F media were mixed with one volume of fresh F medium and the final working conditioned media was supplemented with 5 μM Y-27632.

Strigolactone analogs, MG132 (Tocris), Olaparib and veliparib (Selleckchem) and Mitomycin C (ENZO) were dissolved in DMSO. Doxorubicin (Sigma) was dissolved in ethanol. Irradiation was performed as previously described [51]. Transfections of the I-SceI expression vector (pCBA*Sce*), empty vector (pCAGGS) or positive control (NZEGFP) were performed using Effectene (Qiagen) according to the manufacturer's protocol. For BRCA1 overexpression, U2OS cells were infected with Ad-BRCA1 as previously described [61].

### Cell cycle and apoptosis and caspase assays

Cells treated with vehicle or SLs were harvested, washed twice with 1 × PBS and then fixed in chilled ethanol (70%; v/v in PBS) with gentle vortex mixing. To determine their DNA contents, cells were stained with 40 μg/ml propidium iodide (PI) and analyzed using a FACSCalibur flow cytometer and CellQuest analysis software (Becton Dickinson, San Jose, CA). For apoptosis, cells were harvested and resuspended in 1 × Annexin V Binding Buffer (BD Biosciences, San Jose, CA, USA) prior to the addition of 2 μL FITC-Annexin V (BD Biosciences). Cells were stained with PI (40 μg/mL) and then analyzed by flow cytometry using a BD FACSCalibur flow cytometer. Cells were considered apoptotic if they were Annexin V+/PI− (early apoptotic) or Annexin V+/PI+ (late apoptotic). Each analysis was performed using at least 20,000 events.

### Caspase assay

U2OS cells were treated with 10 ppm of MEB55 for the indicated durations. Samples were trypsinized and seeded (10,000 cells/well) into a white-bottom 96-well plate (100 μL/well) and incubated with equivolume Caspase 3/7-Glo reagents for 30 min at RT. Luminescence was measured on a plate reader (Glomax, Promega). The absolute value of luminescence units was taken by subtracting blank wells of medium alone. The experiment was repeated at least three times and results represent the mean ± SD.

### Metaphase spread

U2OS cells were treated with vehicle or 10 ppm of MEB55 or ST362 for 5 hr. Colcemid (0.1 μg/ml) was added to the medium 1 hr before harvesting. Cells were trypsinized and cell pellets were washed with 1x PBS prior to the addition of hypotonic solution (0.8% sodium citrate). Cells were fixed in Carnoy's fixative (3:1 absolute Methanol: glacial acetic acid) and then dropped onto glass slides, air-dried and stained with 4′,6-diamidino-2-phenylindole (DAPI). Slides were imaged on the Zeiss Axiovert 200M microscope. Averages of chromosome counts of 20 cells per treatment were counted and statistically analyzed by an unpaired t­-test compared to the untreated samples. FANCC cells were treated with 300 nM Mitomycin C or 10 ppm ST362 and MEB55 for three days and metaphase spreads were prepared according to the protocol previously described by Oostra AB, et al. [62].

### Comet assay

The neutral Comet assay selectively tests for double-strand breaks and was carried out using the Trevigen CometAssay Kit (Gaithersburg, MD) as previously described [63]. Following 4 hr treatment with vehicle or 10 ppm MEB55, cells were harvested in ice-cold PBS, mixed with low melting agarose, and plated onto Comet slides at 4°C. Slides were then placed in lysis solution overnight prior to electrophoresis in TBE buffer for 30 minutes at 22V. Slides were washed with water and 70% ethanol and placed in an oven to dry. Comets were visualized following SYBR green staining under a fluorescent microscope at 494 nm. Analysis of the slides was done using the CometScore program (TriTek, Sumerduck, VA). Tail moment (calculated by CometScore) was used to quantify DNA damage.

### Immunoblotting and Immunoprecipitation analysis

Immunoblot analysis was carried out as previously described [7]. Whole cell lysates were prepared and protein concentration was determined using the BCA Protein Assay (Pierce). Immunoblots were performed by incubation with the appropriate antibodies (Supplementary Table 1) for either 2 hr at room temperature or overnight at 4°C. Secondary antibodies were incubated for 30 min at room temperature, and proteins were visualized with West Pico Stable (ThermoScientific). For immunoprecipitation, U2OS cells were treated with 10 μM MG132 for 1 hr prior to treatment with either vehicle or 10 ppm of MEB55 for an additional 4 hr. Three mg of whole cell extracts from vehicle and MEB55-treated cells were incubated with anti-RAD51 polyclonal and protein A/G beads and analyzed by immunoblot with either a monoclonal antibody against ubiquitin or against RAD51. Antibodies used in this study are described in Table S1.

### Immunofluorescence analysis

Cells were seeded on coverslips 24 hr before treatment with SLs. At the indicated times, cells were treated with 0.7% Triton X-100 in Cytoskeletal buffer (10mM Hepes pH 7.0, 100mM NaCl, 300mM Sucrose, 3mM MgCl2, 0.7% Triton X-100), then fixed with 4% paraformaldehyde in PBS. The fixed cells were permeabilized using 0.1% Triton X-100 in PBS for 15 min followed by blocking with 10% goat serum and then incubation with primary antibodies. The bound antibodies were revealed by Goat-anti-mouse IgG coupled to Alexa Fluor 488 (Life Technologies) and goat anti-rabbit coupled to Alexa 594 (Life Technologies). After washes, the coverslips were mounted to slides using Vectashield (Vector Laboratories).

### Homology-directed repair assay

Homologous recombination was measured in DR-GFP-U2OS cells as previously described [42]. Cells were transfected with pCBA*Sce*I (pCBAS), an empty vector (pCAGGS) or with pNZF-GFP as a positive control for GFP-positive cells. After 24 hr, cells were treated with different concentrations of MEB55 for an additional 24 hr and processed for flow cytometric analysis. For each analysis, 1×10^4^ cells were analyzed. Each data point represents the mean ± SD from three independent experiments.

### Non-homologous end joining (NHEJ) assay

The effect of SLs on NHEJ was analyzed *via* a U2OS EJ5-GFP reporter system [44]. Cells were transfected with pCBA*Sce*, an empty vector, pCAGGS, as a negative control or full-length GFP expression construct (NZF-GFP) as a positive control for GFP+ cells. On the next day, cells were treated with the indicated concentrations of ST362 and MEB55 and after an additional 24 hr, cells were processed for flow cytometric analysis. For each analysis, 1×10^4^ cells were collected. Each data point represents the mean ± standard deviation from three independent experiments.

### Topoisomerase-I-mediated DNA cleavage reactions

Human recombinant Top1 was purified from baculovirus as previously described [47]. DNA cleavage reactions were prepared as previously reported with the exception of the DNA substrate [64]. Briefly, a 117-bp DNA oligonucleotide (Integrated DNA Technologies) encompassing the previously identified Top1 cleavage sites in the 161-bp fragment from pBluescript SK(−) phagemid DNA was employed. This 117-bp oligonucleotide contains a single 5′-cytosine overhang, which was 3′ -end-labeled by fill-in reaction with [^32^P] dGTP in React 2 buffer (50 mM Tris-HCl, pH 8.0, 100 mM MgCl2, 50 mM NaCl) with 0.5 units of DNA polymerase I (Klenow fragment, New England BioLabs). Unincorporated [^32^P] dGTP was removed using mini Quick Spin DNA columns (Roche, Indianapolis, IN), and the eluate containing the 3′ end-labeled DNA substrate was collected. Approximately 2 nM of radiolabeled DNA substrate was incubated with recombinant Top1 in 20 μL of reaction buffer [10 mM Tris-HCl (pH 7.5), 50 mM KCl, 5 mM MgCl2, 0.1 mM EDTA, and 15 μg/mL BSA] at 25°C for 20 min in the presence of various concentrations of compounds. The reactions were terminated by adding SDS (0.5% final concentration) followed by the addition of two volumes of loading dye (80% formamide, 10 mM sodium hydroxide, 1 mM sodium EDTA, 0.1% xylene cyanol, and 0.1% bromophenol blue). Aliquots of each reaction mixture were subjected to 20% denaturing PAGE. Gels were dried and visualized using a phosphoimager and ImageQuant software (Molecular Dynamics). For simplicity, cleavage sites were numbered as previously described in the 161-bp fragment [48].

### Ethidium bromide displacement assay

EtBr is a well-known DNA intercalator, and its fluorescence intensity increases considerably after binding with DNA due to intercalation. The EtBr displacement assay was carried out as previously described [45]. Briefly, binding of an agent to DNA would displace the intercalated EtBr and subsequently quench the fluorescence caused by the EtBr-DNA complex. Before measurement, DNA (20 μg/mL) was incubated with EtBr (0.8 μg/mL) for 1 hour. Different concentrationss of SLs were then titrated into the DNA-EtBr solution. The fluorescence intensity of samples was excited at 525 nm, and the fluorescence was measured at 590 nm at a temperature of 25°C using a GloMax+ microplate reader (Promega). Sample fluorescence was determined after subtracting the baseline fluorescence of EtBr in the absence of the DNA. The experiment was repeated three times and results represent mean ± SD.

### XTT viability assay

Cells were seeded into a 96-well plates at 2500 cells per well in triplicates. On the following day, cells were treated with the indicated final concentrations of SLs or vehicle alone. Cells were incubated for 3 days, at which time XTT (2, 3,-bis(2-methoxy-4-nitro-5-sulfophenyl)-5-[(phenylamino)-carbonyl]-2H-tetrazolium inner salt) reduction was used to quantify viability according to manufacturer's instructions (ATCC). Absorbance was recorded by Glomax-Multi Detection plate reader (Promega) at 450 nm with 750 nm of reference wavelength and cell survival was analyzed as previously described [7]. Graph is representative of the mean ± SD of at least three independent experiments.

### Crystal violet growth assays

U2OS cells were seeded at 2,500 cells per well in 6 replicates in 96-well plates. After 24 hr, cells were treated with MEB55, ST362 or vehicle (DMSO) at the indicated doses (1.25 to 10 μM) and in combination with Doxorubicin (Sigma). After 72 hr, cells were fixed and stained with crystal violet solution (0.5% crystal violet and 25% methanol in dH_2_O). Following extensive washing with distilled water, plates were air dried overnight. Sodium citrate solution (0.1 M) was used to solubilize bound crystal violet and optimal densities were measured at 560 nm (Glomax-Multi Detection plate reader, Promega). All survival fractions are relative to vehicle-treated control cells. Graph is representative of the mean ± SD of at least three independent experiments.

### Drug interactions

DOX and SLs drug mixtures for calculation of combination index (CI) values were prepared by twofold serial dilutions of working concentrations for doxorubicin and MEB55. Using non-constant ratios, two concentrations above and below the calculated IC_50_ (corresponding to 0.25, 0.5, 1, 2 and 4 times the IC_50_) were added to cells in XTT assay. Each concentration was tested in 6 replicates and the results were confirmed in at least three independent experiments. For interaction between PARP inhibitors and MEB55, different concentrations of olaparib or veliparib were tested in the presence of MEB55 IC_50_ and IC_25_. Experiments were repeated at least three times with six replicates each. The interaction between PARP inhibitors and MEB55 was analyzed according to Kern, et al. [50].

### Statistical analysis

Results are presented as mean ± SD of replicate analyses and are either representative or inclusive of at least three independent experiments. All statistical analyses were performed using two-tailed Student's *t*-tests in GraphPad PRISM 6 software. In all figures, significant differences between specified pair of conditions, as judged by *t*-test, are indicated using asterisks (*0.01 < *P* < 0.05; **0.001 < *P* < 0.01; ***0.0001 < *P* < 0.001). IC_50_ doses for SLs were calculated by interpolation of the sigmoidal dose response curves (Graphpad Prism 6.0 software).

## SUPPLEMENTARY INFORMATION TABLES AND FIGURES


