# Supplementary material for: Analogs of the novel phytohormone, strigolactone, trigger apoptosis and synergize with PARP inhibitors by inducing DNA damage and inhibiting DNA repair
Source: Oncotarget. 2016 Feb 16;7(12):13984–4001. doi: 10.18632/oncotarget.7414 (PMC4924693; doi:10.18632/oncotarget.7414)
Supplement: Supplementary file 1 [file oncotarget-07-13984-s001.pdf]

Analogs of the novel phytohormone, strigolactone, trigger apoptosis and synergize with PARP inhibitors by inducing DNA damage and inhibiting DNA repair

Supplementary Information

Materials and Methods:

1) Table 1: Antibodies used

Antibody	Clone	Source
Actin-HRP	HRP-60008	Proteintech
ATM	2CI	SCBT
p-ATM	10H11.E12 PE	Cell Signaling
p-ATR	S428	Cell Signaling
Chk1	G-4	SCBT
p-Chk1	#2341	Cell Signaling
p-Chk2	#2661	EMD-Millipore
H2A	OP92	EMD-Millipore
γH2AX	JBW301	EMD-Millipore
53BP1	A300	Bethyl
PARP1	#9542	Cell Signaling
RAD51	H92	SCBT

2) Results

Table S2: C-Index Data for Non-Constant Combo: m-DOX (DOX+MEB55)

**IC50**	**Dose DOX (µM)**	**Dose MEB55 (ppm; [µM])**	**Effect**	**CI**
x 4	6.88	10.8 [32.4]	0.11	1.14237
x 2	3.44	5.4 [16.2]	0.268	1.2758
IC50	1.72	2.7 [8.10]	0.33	0.79591
IC25	0.86	1.35 [4.05]	0.55	0.78785
IC13	0.43	0.675 [2.02]	0.79	0.92621

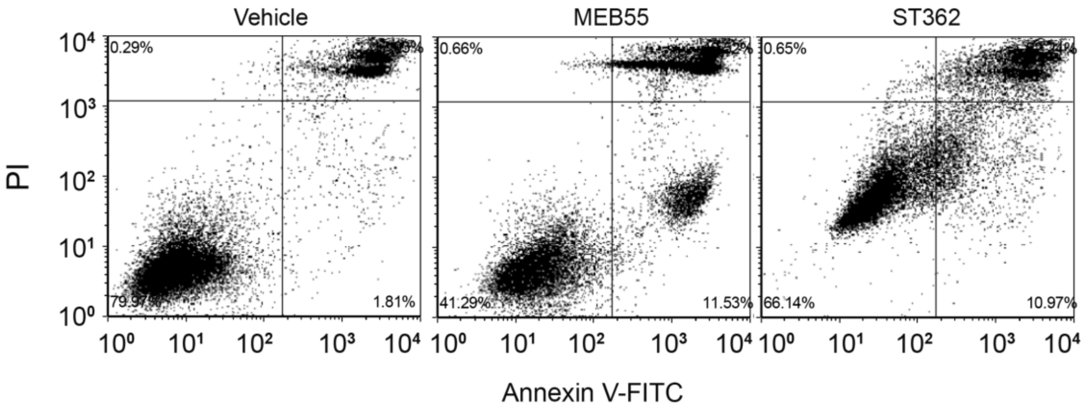

Supplementary Figure S1: U2OS cells were stained with Annexin V and propidium iodide and apoptosis was analyzed using flow cytometry.

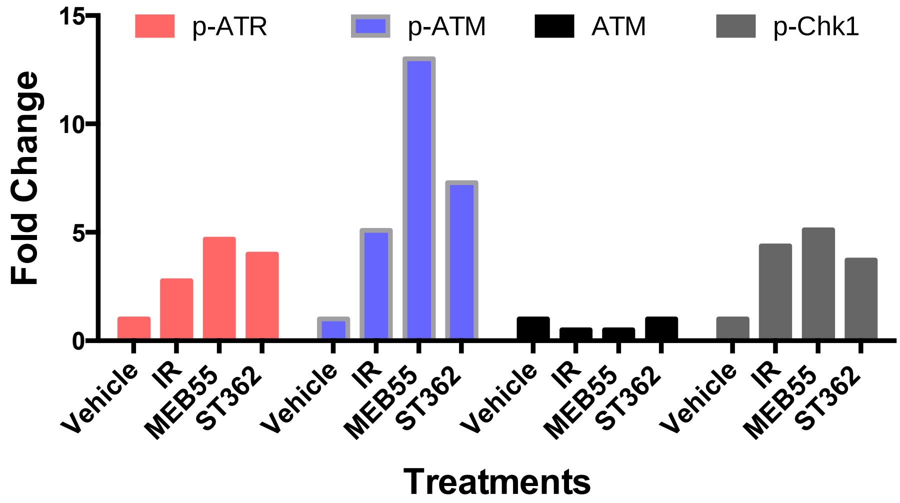

Supplementary Figure S2: Results of several immunoblot analysis were quantified using the free software ImageJ. Bands' intensity was normalized to loading control and to vehicle-treated samples for each protein analyzed.

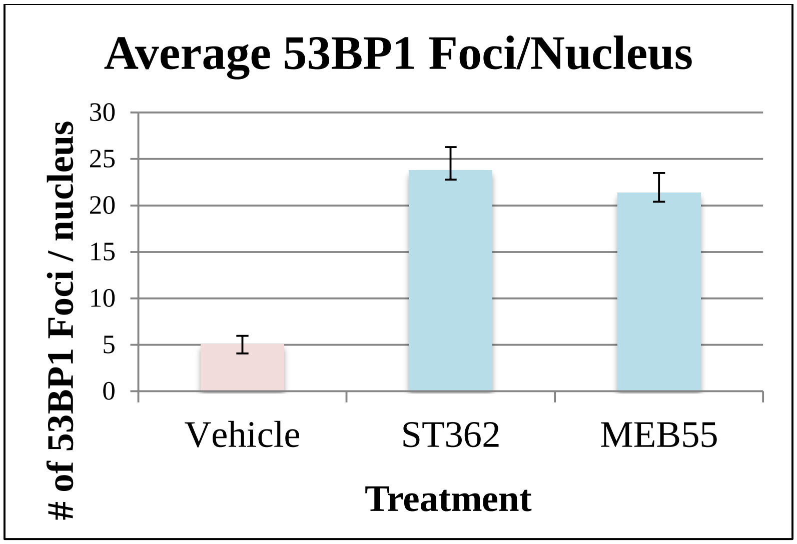

Supplementary Figure S3: 53BP1 foci were counted in each of the three treatment groups. Values represent means ± SEM. (n= 250 cell nuclei for each group). There were significantly more foci in both MEB55 and ST362-treated cells versus vehicle-control cells as determined by a student's *t*-test (p <0.001). There was no significant difference between ST362 and MEB55-treated cells in terms of number of foci or percentage of cells with >10 foci (p = 0.484).

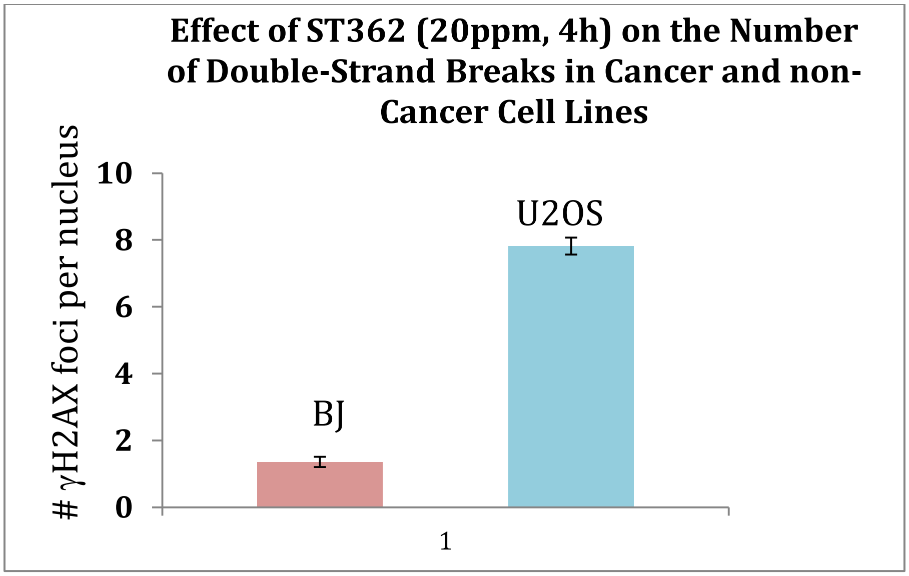

Supplementary Figure S4: U2OS cells were selected at 40x magnification based solely on DAPI stain fluorescence to allow for non-biased selection, then photographed with the GFP filter to image the number of yH2AX foci per cell nucleus. Images were processed with CellProfiler open source software*. Values represent means ± SEM. For BJ nuclei, n=259. For U2OS nuclei, n=324. P<0.0001 as determined by Student's *t* test.

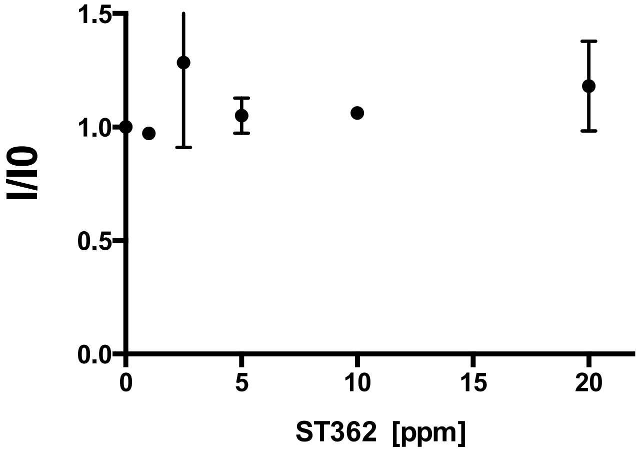

Supplementary Figure S5: Circular DNA was incubated with EtBr at 0.8ug/mL for 1 hr before the indicated concentrations of ST362 were added. The fluorescence intensity of the samples was excited at 525 nm, and measured at 590 nm.

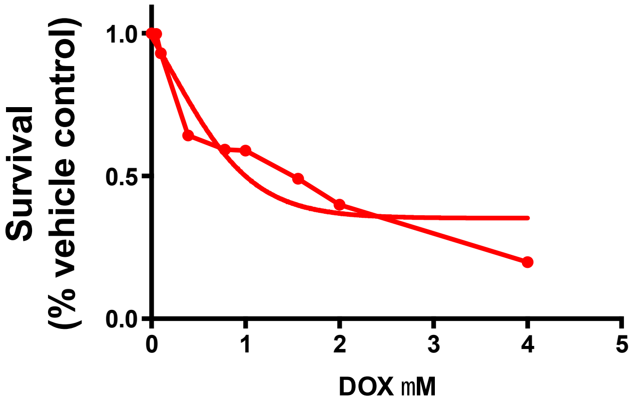

Supplementary Figure S6: U2OS cells were seeded into 96 well plates (2500 cells/well) and on the following day, cells were treated with the indicated doses of DOX. Cell viability was assayed after 4 days by XTT. Graph is representative of mean of three independent experiments and three replicates in each experiment. The IC_{50} was calculated following non-linear regression analysis using GraphPrism 6.0

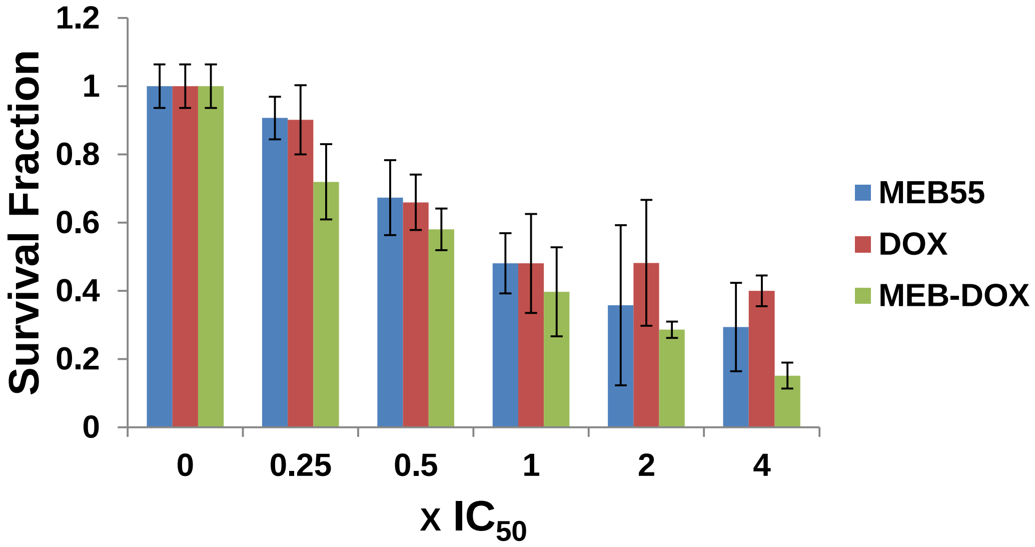

Supplementary Figure S7: DOX-MEB55 Treatment combination. U2OS cells were seeded into 96 well plates (2500 cells/well) and on the following day, cells were treated with combination of DOX and MEB55. Combinations span 2 and 4 fold increase of the IC50 concentration and 2 and 4 fold decrease of the IC50 concentrations. Cell viability was assayed after 4 days by XTT. Graph is representative of mean of three independent experiments and three replicates in each experiment.

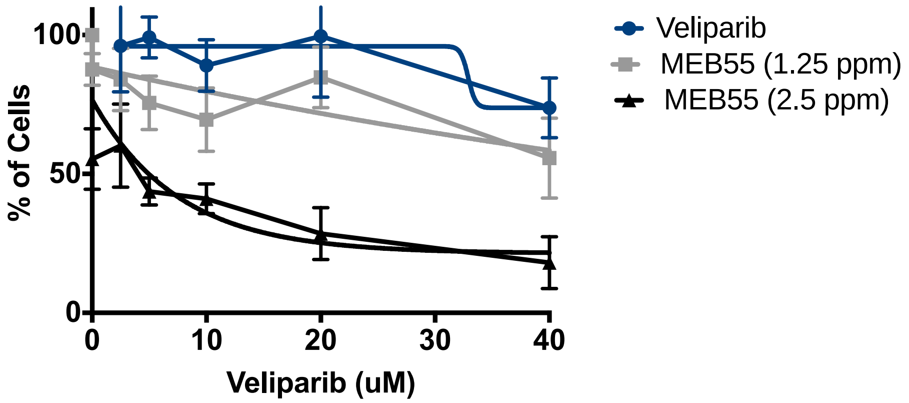

Supplementary Figure S8: U2OS cells were seeded into 96-well plates (2500/well) in 6 replicates and their growth in response to 96 hr treatments with different concentrations of MEB55 and veliparib relative to vehicle-treated (DMSO) cells was analyzed by crystal violet assay. Graph is representative of mean ± SD from at least three independent experiments.

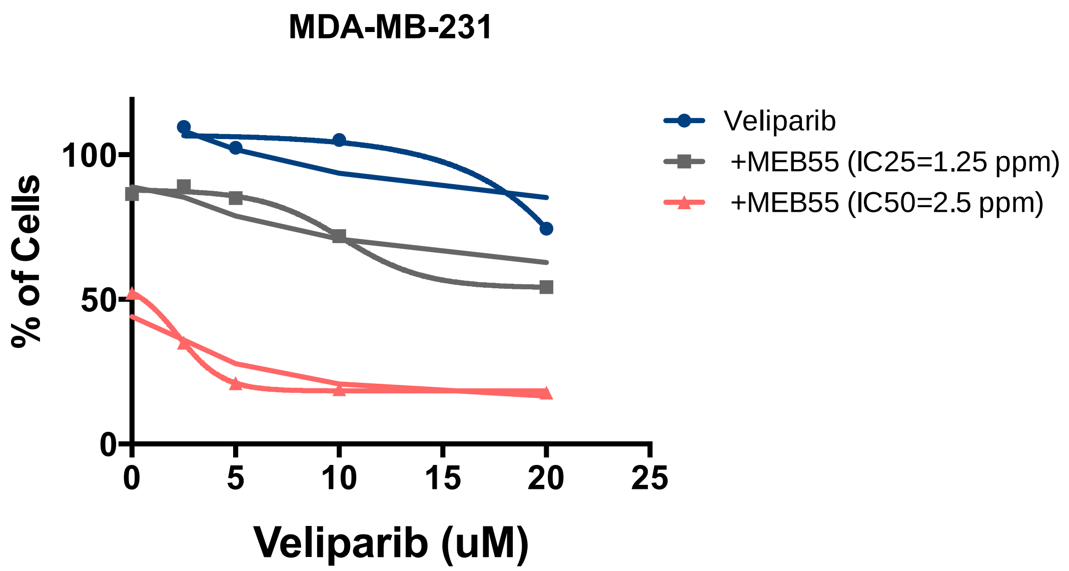

Supplementary Figure S9: MDA-MB-231 cells were seeded into 96-well plates (2500/well) in 6 replicates and their growth in response to 96 hr treatments with different concentrations of MEB55 and veliparib relative to vehicle-treated (DMSO) cells was analyzed by crystal violet assay. Graph is representative of mean ± SD from at least three independent experiments.
